# NRF2-ome: An Integrated Web Resource to Discover Protein Interaction and Regulatory Networks of NRF2

**DOI:** 10.1155/2013/737591

**Published:** 2013-04-17

**Authors:** Dénes Türei, Diána Papp, Dávid Fazekas, László Földvári-Nagy, Dezső Módos, Katalin Lenti, Péter Csermely, Tamás Korcsmáros

**Affiliations:** ^1^Department of Genetics, Eötvös Loránd University, Pázmány P. s. 1C, H-1117, Budapest, Hungary; ^2^Department of Medical Chemistry, Faculty of Medicine, Semmelweis University, Tűzoltó u. 37-47, H-1094, Budapest, Hungary; ^3^Department of Morphology and Physiology, Faculty of Health Sciences, Semmelweis University, Vas u. 17, H-1088, Budapest, Hungary

## Abstract

NRF2 is the master transcriptional regulator of oxidative and xenobiotic stress responses. NRF2 has important roles in carcinogenesis, inflammation, and neurodegenerative diseases. We developed an online resource, NRF2-ome, to provide an integrated and systems-level database for NRF2. The database contains manually curated and predicted interactions of NRF2 as well as data from external interaction databases. We integrated NRF2 interactome with NRF2 target genes, NRF2 regulating TFs, and miRNAs. We connected NRF2-ome to signaling pathways to allow mapping upstream NRF2 regulatory components that could directly or indirectly influence NRF2 activity totaling 35,967 protein-protein and signaling interactions. The user-friendly website allows researchers without computational background to search, browse, and download the database. The database can be downloaded in SQL, CSV, BioPAX, SBML, PSI-MI, and in a Cytoscape CYS file formats. We illustrated the applicability of the website by suggesting a posttranscriptional negative feedback of NRF2 by MAFG protein and raised the possibility of a connection between NRF2 and the JAK/STAT pathway through STAT1 and STAT3. NRF2-ome can also be used as an evaluation tool to help researchers and drug developers to understand the hidden regulatory mechanisms in the complex network of NRF2.

## 1. Introduction

NRF2 (NF-E2-related factor 2, NFE2L2) transcription factor is the master controller of oxidative and xenobiotic stress responses [1]. Function of NRF2 influences oxidative stress-related physiologic and pathologic processes such as carcinogenesis, inflammation, and neurodegeneration [2]. Upon oxidative stress, NRF2 enters the nucleus and activates the expression of antioxidant-responsive-element- (ARE-) dependent genes to maintain cellular redox homeostasis [3]. Activation of NRF2 is a well-known response to environmental and endogenous stresses. NRF2 activation is mainly regulated by signaling processes and pathways (e.g., MAPKs and PI3K) that target NRF2 or its cytoplasmic repressor, KEAP1 [3, 4].

While there are nearly 3000 articles for the keyword ‘‘NRF2” in PubMed (as of January 2013), major network resources (BioGRID, MINT, STRING, HPRD and InnateDB) contain only a few dozen NRF2 interactors. Prompted by the lack of a systems-level NRF2-related resource, we recently collected the literature information on NRF2 interacting proteins and regulated genes, as well as predicted novel NRF2 interactors and regulators [5]. We also imported NRF2 regulating transcription factors (TFs) and miRNAs from major network resources. The NRF2 interactome and regulome datasets allowed us to examine fine-tuning autoregulatory loops of NRF2 and to identify multifunctional proteins interacting with NRF2 [5]. However, the former dataset [5] is stored in more than 16 separate datasheet tables, containing minimal information on the proteins (only the short name of a protein with one UniProt ID) and no links to web resources or other datasheets. This format makes further analysis difficult for several reasons. (1) Search in the datasheets requires the canonical short name of a protein though each protein has several synonyms. (2) The separate datasheets neither allow any global search for a given protein, nor provide a uniform data collection about an NRF2 interactor. (3) Only experts with computational background could visualize and perform network analysis with the datasets. Importantly, the formerly published datasheets [5] contain mostly NRF2-centric (i.e., star-like) interactions and regulatory information and lack important cross-regulatory connections between NRF2 interactors and other TFs or signaling proteins that are indirectly connected to NRF2.

Here, we report the development of a user-friendly web resource to analyze systems-level data on NRF2, which we hope will provide a help for researchers working with NRF2 or NRF2-related processes. Going beyond the creation of a database based on the datasheets on NRF2 interactome and regulome [5], we extended this database with further protein-protein and regulatory information. We also included signaling pathway data from SignaLink 2, a signaling network resource we previously developed [6, 7]. This process extended the number of protein-protein interactions from the previously published [5] 311 to 13,053 and the regulatory connections from 8,833 to 22,095. Finally, we created a novel website (http://nrf2.elte.hu/) to provide an easy-to-use graphical interface allowing users to browse or download the NRF2-ome resource. 

## 2. Materials and Methods

### 2.1. Compilation of NRF2-ome Resource

**Figure 1:**
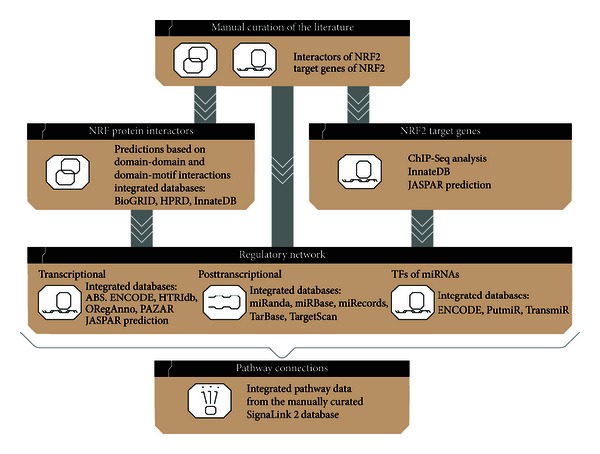
The workflow of the compilation process of NRF2-ome. NRF2-ome is based on the manually curated collection of first neighbor interactors and target genes of NRF2 [5]. The manual curation was extended and complemented with the integration of external databases and predictions on further NRF2 interactors and target genes. The network of TF and miRNA regulators was built upon this interactome and regulome. At this phase, the TFs of miRNAs were also included. Finally, we integrated upstream connections to manually curated signaling pathway from the SignaLink 2 resource.

The NRF2-ome contains a manually curated core interactome, which was extended with further protein-protein interactions (PPIs) as well as with transcriptional and posttranscriptional regulatory components and interactions. In the following, we list the compilation process of the NRF2-ome resource (Figure 1).

The starting point of NRF2-ome was a set of 112 interactions between 84 NRF2 interactor proteins that we previously developed by manual curation of the literature [5]. All these interactions were found in human cells and further information about the interactions (e.g., direction, and the literature reference) were already listed. To extend this information, we used experimentally verified interactions from PPI databases and *in silico *predictions to create the network of first neighbors of NRF2. We used the following PPI databases: InnateDB [8], HPRD [9], and BioGRID [10]. InnateDB and HPRD contain mostly curated interaction data from small- and medium-scale studies, while BioGRID also contains high-throughput studies. For additional enrichment of the NRF2-ome resource, we predicted novel interactions based on protein structure data. We used domain composition data from PFAM and domain-domain interactions data from DOMINE [11, 12] to predict undirected PPIs based on domain-domain interactions. To predict directed PPIs, we used domain-motif-interactions, retrieved from the ELM Server [13]. We used only those domain-motif-based PPIs for which ELM's Structure Filter cut-off value was above the default.

Next, we integrated regulatory information for NRF2 and for the already included first neighbors of NRF2. To identify TFs that regulate NRF2 or its first neighbors, we imported TF-target gene interactions from the following databases: ABS, ENCODE, HTRIdb, JASPAR, and ORegAnno [14–18]. To list the target genes of NRF2, we used the previously curated data of the literature [5], as well as integrated data from InnateDB [8], two ChIP-Seq profiling studies [19, 20], and JASPAR-based predictions [8]. If an interaction was found in multiple resources or methods, then we listed all versions, offering a comprehensive view. We also integrated posttranscriptional regulatory interactions (i.e., miRNAs that can regulate NRF2 or its first neighbors) by manual curation and from miRBase, TarBase, Miranda, TargetScan, and miRecords resources [21–25]. To list those TFs that can regulate NRF2-regulating miRNAs, we used data on the transcriptional regulation of miRNAs from ENCODE, PutMir, and TransMir resources [18, 26, 27].

Finally, we used SignaLink 2, a signaling network resource we recently developed [6, 7], to connect all of the already included proteins to signaling pathways. SignaLink 2 contains 7 major signaling pathways: RTK (receptor tyrosine kinase), TGF-*β* (transforming growth factor beta), WNT/Wingless, Hedgehog, JAK/STAT, Notch, and NHR (nuclear hormone receptor) [6]. Integration of signaling pathways to NRF2-ome allows the mapping upstream components of NRF2 and other TFs, which regulate NRF2 or its first neighbors. As a final step, we used BioGRID [10] and HPRD [9] resources again to include further PPIs known between all the inserted components (e.g., PPIs between TFs, or between signaling pathway components and NRF2 interactors).

### 2.2. Database Implementation and Structure

NRF2-ome stores data in a MySQL database, which is connected to the webpage by an interface written in PHP. On the client side, the webpage uses jQuery to offer a high interactivity. It loads data asynchronously by small http requests, making possible to efficiently browse through hundreds of interactions. Data can be exported and downloaded in various formats: CSV, BioPAX, PSI-MI TAB, PSI-MI XML, SBML, and Cytoscape's CYS format. The download page offers several options to customize the network to be the downloaded; user can select desired interaction types (e.g., PPIs and transcriptional regulation) and filter the interactions by sources. There is an option to separate experimentally verified and predicted interactions. The customized network files are generated after the selection, by an export module running in the background, implemented in Python. This process can take few minutes. Then, for each download, the database generates a URL, where users can access the data for 14 days. Optionally, users can provide their e-mail addresses to which files smaller than 10 MB will be e-mailed. The whole dataset is also available as a standard SQL dump, so that any complex query or modification can be applied using SQL statements.

**Figure 2:**
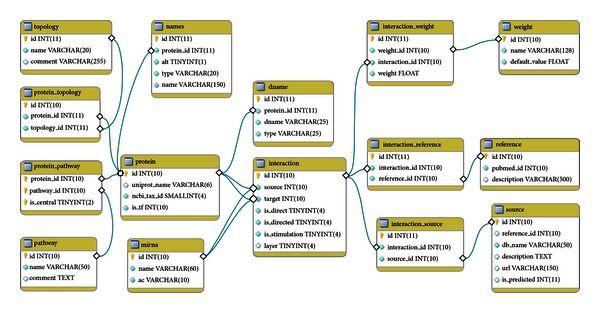
The SQL database scheme of the NRF2-ome resource. See the main text for details.

The core of NRF2-ome database is the interaction table. In the interaction table *source* and *target* fields are integers pointing to the primary keys of protein or mirna tables. The *layer* field denotes the type of the interaction, and its value determines whether the source or the target refer to a protein or miRNA. The meanings of the values in the layer field are the following: 1: PPIs in the interactome of NRF2; 4: TF-target interactions; 5: miRNA-mRNA interactions; 6: PPIs in the signaling pathways, imported from SignaLink 2; 7: TF-miRNA connections (values 2 and 3 are not functional in the current version of the database). Each interaction has three main attributes: *is_directed* (0: undirected; 1: directed; 2: direction is predicted), *is_direct* (0: indirect; 1: direct), and *is_stimulation* (0: unknown; 1: stimulation, -1: inhibition). In addition, interactions have one or more sources. Sources are listed in the source table, and the interaction_source table contains their assignment to the interaction table. Manually curated interactions have the literature references, contained by the interaction_reference table. In the interaction_reference table, articles are identified by their Pubmed IDs. Most of the interactions have confidence scores. These are stored as float values in the interaction_weight table, while the different types of scores are listed in weight table. Components of the NRF2-ome are listed in the protein and mirna tables. The protein table contains the *uniprot_name* field, which is unique, and it contains the UniProt accession number of proteins. All records imported from other databases as well as protein names from articles have been mapped to their primary UniProtKB ID. Proteins may have signaling topological properties and pathway assignments, available in protein_topology and protein_pathway tables. In the mirna table, we used miRBase AC and miRNA name to identify miRNAs.  Figure 2 shows the scheme of the SQL database.

## 3. Results and Discussion

### 3.1. The NRF2-ome Database

**Figure 3:**
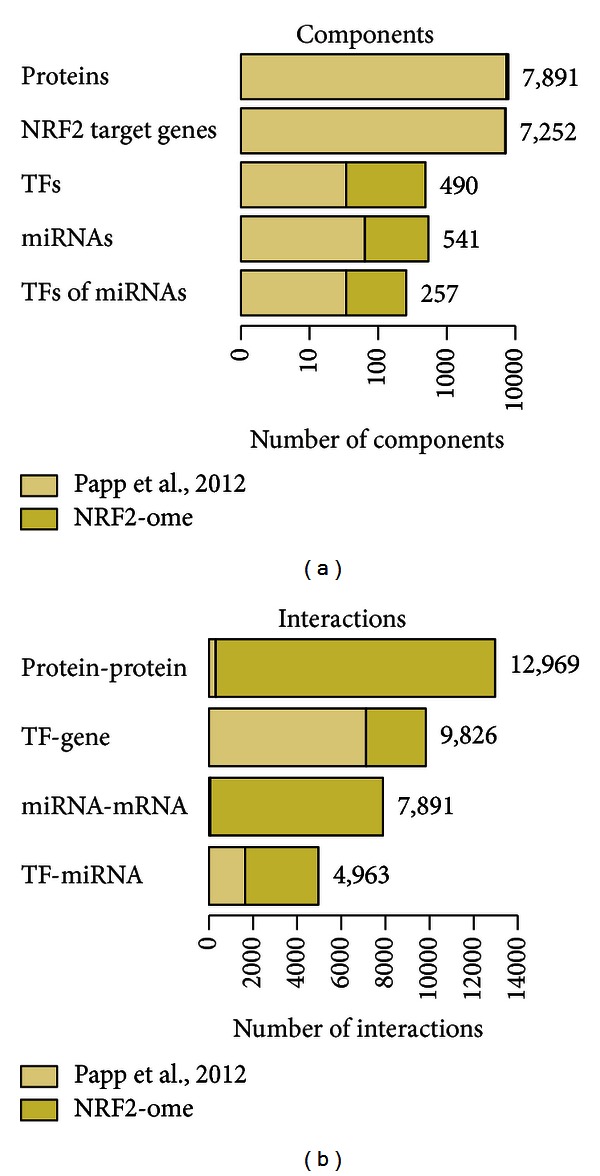
Number of components and interactors in the NRF2-ome database. For each category, darker color indicates the improvement of the dataset compared to the dataset of our earlier publication [5] shown with light color. (a) The number of components (proteins, target genes, TFs, miRNAs, and miRNA regulating TFs) in the NRF2-ome database. (b) The number and type of interactions in the NRF2-ome database. Note the small portion of protein-protein interactions (311) and miRNA-mRNA interactions (64) in the earlier publication [5]. This improvement transforms the originally NRF2-centric (i.e., star-like) dataset into a densely connected network resource. We also note that the earlier publication [5] contained 7,469 target gene IDs, while NRF2-ome contains 7,252 IDs because since then, some of the IDs have been deleted or merged in the Uniprot resource. Thus, there is a slight decrease in the number of target genes in NRF2-ome, but this decrease is coupled with more reliable IDs.

The NRF2-ome database contains interactors and regulators of NRF2, their interactions to NRF2, and physical as well as regulatory interactions between them. Altogether, the NRF2-ome database contains 7,777 proteins and 35,967 interactions (Figure 3). From the 7,777 proteins, 227 are directly interacting with NRF2, 45 are TFs directly regulating NRF2, while 165 TFs are regulating miRNAs capable to downregulate NRF2. 7,252 proteins in the NRF2-ome database are encoded by genes regulated by NRF2. Interestingly, there are only 108 proteins that are both interactors and target genes of NRF2. We also integrated signaling pathway data and found that from the 7,777 proteins, 591 are involved in signaling. As a more indirect connection, we found 51 signaling pathway proteins interacting with NRF2, 163 pathway proteins binding to NRF2 interactors and 8 pathway proteins affecting TFs regulating NRF2. We note that the NRF2-ome database also contains pathway connections through second neighbor interactors.

There are four types of interactions in the NRF2-ome database: (1) protein-protein interactions; (2) TF-target gene regulatory connections; (3) miRNA-mRNA regulatory interactions; (4) TF-miRNA regulatory connections.  Figure 3(b) shows the number of interactions by types in the NRF2-ome database. For all four categories, NRF2-ome contains both experimentally verified and predicted connections. As NRF2-ome distinguishes between these two evidence types and also interactions are stored with their original sources, users can examine interactions separately according to their requirements.

### 3.2. The NRF2-ome Website

We developed a user-friendly web interface for NRF2-ome that can be accessed at http://nrf2.elte.hu/. The aim of the website is twofold. (1) It gives an interactive opportunity to browse the NRF2-ome database and use hyperlinks to other web resources and Pubmed abstracts. (2) It provides an easy-to-use download interface, where users without computational background can select data from the content of the NRF2-ome database. The search field available on the main page autocompletes the queried terms to facilitate users' search. The search engine of NRF2-ome understands many database IDs, accession numbers, and protein names based on the mapping table of UniProt [28]. If the search term is ambiguous, users can manually select the needed item from a list.

**Figure 4:**
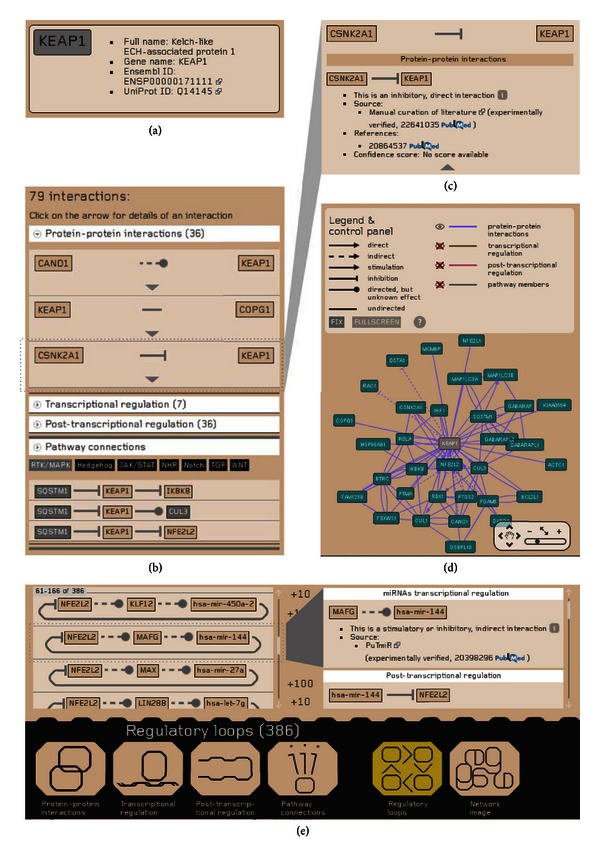
Combined snapshots of the NRF2-ome web resource. (a)–(d) The protein datasheet page of KEAP1. (a) The header contains the full name, gene name, UniProt ID, and Ensembl ENSP ID of KEAP1. (b) The interaction list of KEAP1 shows the interactions by types and also the ‘‘Pathway connections” section, which shows one- or two-step-long connections to signaling pathway member proteins. Detailed information on the interactions can be accessed by clicking on the triangles appearing below each interaction. (c) The slide-down box contains sources, references, and confidence scores of the interactions. (d) The protein datasheet page also contains an interactive visualization of the network of KEAP1. (e) NRF2 has a special datasheet page at the website, which makes interaction browsing easier for the users. On the bottom of NRF2 datasheet, page users can choose the interaction types to be shown as well as the regulatory loops involving NRF2. Here, all arrows symbolizing interactions are clickables, to get the same information about the interaction as on the protein datasheet. We show the details of the regulatory loop involving MAFG and mir-144 as an example.

If the search is successful, a datasheet of the selected protein or miRNA will be shown (Figures 4(a)–4(d)). The header of the protein datasheet shows the full name, gene name, Ensembl protein ID, and UniProtKB AC of the protein. Below the header, an interactive interaction list is shown to present the first neighbor interactors of the protein. All details of an interaction can be examined here, including the interaction properties (direct/indirect, directed/undirected, stimulatory/inhibitory, and predicted/experimentally verified) and the Pubmed links to the external source from where we integrated the given interaction, and Pubmed links to the original paper used as a reference. At the bottom of the page, we present a ‘‘Pathway connections” section. Here, users can examine connections of the given protein and members of a signaling pathway interactively selected by the user. We distinguish between upstream and downstream pathway interactors (i.e., interactors affecting the given protein or interactors affected by the given protein, resp.).

On the right side of the protein datasheet page, an interactive network image is presented, using the Cytoscape Web embedded flash application [29]. Clicking on the nodes or edges brings the user to the datasheet page of the selected protein, miRNA, or interaction. The network view can be enlarged to full screen mode. By default, only manually curated and integrated PPIs are shown. Further interactions can be turned on by the user; however, in case of too many nodes and edges, the limitations of the flash application may make the visual experience uncomfortable. In this case, the webpage gives a warning message before visualizing the network and recommends the possibility to download a custom network using the download option of the webpage and visualize the network offline.

In case of the NRF2 protein, the website provides a different view because of the high number of interactions (Figure 4(e)). At the datasheet page of NRF2, the interaction types and the network image are shown separately, and the interaction lists are loaded only in small portions to optimize the interactive visual experience. Only in the datasheet page of NRF2 we list regulatory loops. Regulatory loops contain two or three nodes that form positive or negative feedbacks to regulate NRF2. The shorter type of these loops consists a protein interacting with NRF2, or a TF of NRF2, which is regulated reciprocally by NRF2 at the transcriptional level. NRF2-ome contains 24 and 12 such proteins and TFs, respectively. A more loose type of regulatory loops involves miRNAs that repress the translation of NRF2 and a TF regulating the transcription of that miRNA and regulated by NRF2. In NRF2-ome there are 385 such regulatory loops, involving 61 miRNAs and 82 TFs.

The download option is accessible under the ‘‘download” menu of the webpage. The user interface offers an easy way to select the desired parts of the database. The entire database is available as a MySQL dump file. Alternatively, for less-experienced users, we developed a BioMART-like customizable download page, where users can easily select interaction types and file format of the download. A general switch is also available to exclude all predicted interactions. The customized subnetworks can be downloaded in various formats: CSV, BioPAX, SBML, PSI-MI tab or PSI-MI XML, and in a Cytoscape CYS file.

### 3.3. Applications of the NRF2-ome Website

We present three examples to illustrate the applicability of the NRF2-ome website. We show how regulatory loops and pathway connections can point out important novel information about NRF2. Both approaches require the integrated transcriptional and signaling data present in NRF2-ome.

To illustrate the functionality of NRF2 regulatory loops, we investigated the list of miRNA-containing regulatory loops. These loops are formed by NRF2, a target gene of NRF2 that functions as a TF, which regulates a miRNA capable to downregulate the translation of NRF2. First, we selected only those loops, where the effect of the miRNA was experimentally verified. By this, we narrowed the list of miRNAs from 63 to four miRNAs (miR-27a, miR-28, miR-93, and miR-144). These four miRNAs form 20 identical loops with 14 different TFs. We selected the MAFG transcription factor as an example because it was predicted to regulate the expression of two from the four miRNAs (miR-93 and miR-144) [26], and there is experimental evidence that NRF2 positively regulates the expression of MAFG [19] (Figure 4(e)). MAFG and NRF2 proteins form heterodimers to promote gene expression of ARE-dependent genes [3]. However, MAFG homodimers and heterodimers with other small MAF proteins can repress NRF2 transcriptional activity by competing with NRF2 at ARE binding sites [30, 31]. Based on the two regulatory loops involving MAFG and miR-93 or miR-144, here, we suggest an indirect, posttranscriptional negative feedback, where MAFG could downregulate the expression of NRF2.

As we integrated signaling pathway data to NRF2-ome, users can examine the pathway annotations and signaling connections of known and predicted NRF2 interactors or regulators. For this analysis, we selected the JAK/STAT pathway as it has high functional overlap with NRF2. To predict and explain pathway connection between the JAK/STAT pathway and NRF2, we selected and examined two JAK/STAT pathway members: STAT1, a predicted TF of NRF2, and STAT3, a potential interactor of NRF2.

STAT1 is activated in response to interferons (IFN alpha, beta, and gamma) upon viral infection and inflammation [32]. As NRF2 has been found be induced by viral infection and IFN gamma [33, 34], and NRF2 deficient (−/−) mice had more severe injuries upon viral exposure [35], we may hypothesize that STAT1 as a TF can be an important regulator in the antiviral and anti-inflammatory role of NRF2. Further experimental validation should determine the role of STAT1 in the expression of NRF2 upon viral infection.

Another member of the JAK/STAT pathway, STAT3 was predicted to interact with NRF2 based on domain-motif interactions. Though we found no publication that could validate STAT3-NRF2 interaction, we found several pieces of indirect, functional evidence, which support this interaction. Both STAT3 and NRF2 have anti-inflammatory and antiapoptotic functions upon ethanol exposure [36, 37]. In liver cells, ethanol-induced inflammation and apoptosis are repressed by globular adiponectin that increases the production of heme-oxygenase 1 (HO-1) through NRF2 and the IL-10/STAT3 pathway [38, 39]. As HO-1 can also be induced in an NRF2-independent way [38], future experiments will be needed to clarify the role of IL-10 and STAT3 in NRF2-dependent HO-1 induction. In addition, experimental approaches could validate that IL-10 indeed influences the activation of NRF2. It is intriguing to speculate that STAT3 and NRF2 may cooperate to serve as anti-inflammatory and antiapoptotic TFs upon ethanol exposure.

### 3.4. Discussion

In this work, we significantly extended our previously developed NRF2 interactome and regulome dataset [5] and developed it to an easy-to-use systems-level resource. The current database provides 214 manually curated and predicted NRF2 interactors as well as includes interactors and regulators from several external resources covering nearly four times more information than its preliminary version published earlier [5]. The integrated and unified database structure allows the users to search data from different sources in a single resource. Thus, it does not require prior bioinformatics knowledge on the prediction tools and on the structures of the different databases. NRF2-ome also serves as an integrated resource, where protein interaction and regulatory and signaling data can be analyzed simultaneously. The included manual curation was performed in early 2012, and it will be updated regularly (in each 2 to 3 years). In addition, as data in the integrated external resources will probably be extended in the future, we designed the database building scripts that the database can be easily updated from the same resources. We plan to update data from external resources every year.

The NRF2-ome website that we developed to serve as a graphical interface to the NRF2-ome database (http://nrf2.elte.hu/) provides a user-friendly environment to interactively search, browse, or download the database. The UniProt-based, automatic mapping function of the search module allows users to search with different names or database IDs of a protein. On the website, network data on proteins directly or indirectly related to NRF2 can be easily accessed. Hyperlinks to further resources and literature references facilitate the interactive and fast exploration of protein functions. As a unique feature, we show signaling pathway connections to the proteins, including NRF2, other TFs, or first neighbors of NRF2. This option allows mapping upstream components that could directly or indirectly influence NRF2 activity. For NRF2, a specific website function is available, where users can browse regulatory loops containing possible feedback mechanisms that could up- or downregulate the expression of NRF2. After or instead of browsing the website, users can download the NRF2-ome database as a whole or a user-specified part of it. We provide a user-friendly download page allowing researchers without computational background to select and filter the NRF2-ome database. As the download is possible in CSV and Cytoscape formats, users without advanced bioinformatics knowledge can instantly examine and visualize the data of their interest.

As with most bioinformatics resources, the NRF2-ome has several limitations. Many of the predicted regulatory loops may not be functional or may function only under very specific circumstances. Therefore, users should keep in mind that experimental validation is needed to confirm the function of a predicted feedback loop. Due to the high complexity and centrality of NRF2 regulation, it is also possible that in most cases the effect of one miRNA is negligibly low to the overall expression of NRF2. As we have limited information about the effect of most of the transcriptional events (i.e., whether the TF has a stimulatory or inhibitory effect), in most of the cases we cannot distinguish between positive and negative feedbacks.

Keeping in mind the limitations listed before, the NRF2-ome is capable to provide both simple predictions (i.e, list predicted PPIs or regulatory interactions), as well as complex predictions involving TFs, miRNAs, or signaling pathways. To illustrate the applicability of NRF2-ome, we presented examples about MAFG protein containing regulatory loops and raised the possibility of a connection between NRF2 and the JAK/STAT pathway through STAT1 or STAT3. All three examples are predictions; thus, further experimental validation is needed to confirm them. The NRF2-ome can also be used as an evaluation tool to help researchers explain a given expression pattern. A combination of the NRF2-ome network and user-made expression datasets with the Cytoscape application could help to uncover hidden regulatory mechanisms.

NRF2-ome could also be used as a resource for network pharmacology. The medical importance of NRF2 is coming from its involvement in oxidative stress, inflammation, and many age-related diseases, including cancer, neurodegenerative diseases, and diabetes [40, 41]. The NRF2 activators are potential therapies for oxidative stress, inflammation, and chemoprevention [40]. Activation of NRF2 in healthy cells could delay or prevent the onset of some forms of human cancers [41], but its constitutive activation is responsible for acquired chemoresistance in tumor cells [42, 43]. Therefore, investigating the complex interaction and regulatory and signaling network that influence the activation of NRF2 could facilitate novel pharmacological attempts. The NRF2-related regulatory loops and pathway connections listed in NRF2-ome could help the evaluation of drug development failures and guide developers to target proteins with clear effect on NRF2. We hope that NRF2-ome will serve as resource for such attempts and help researchers to identify drug targets that can specifically modify the activity of NRF2.

## 4. Conclusions

We have compiled a network resource, which contains a total of 7,777 manually curated, integrated, and predicted interaction data of NRF2, its first neighbor interactors, its target genes, regulating TFs, and miRNAs, as well as signaling pathways regulating NRF2. The user-friendly website (http://nrf2.elte.hu/) allows researchers without computational background to search, browse, and download the database. NRF2-ome contains integrated information on regulatory loops and pathway connections of NRF2 and its interactors. NRF2-ome is able to provide interesting predictions to be tested experimentally as well as to help researchers to evaluate experimental data or drug treatment outcomes.
